# Network construction and structure detection with metagenomic count data

**DOI:** 10.1186/s13040-015-0072-2

**Published:** 2015-12-12

**Authors:** Zhenqiu Liu, Shili Lin, Steven Piantadosi

**Affiliations:** 1 Samuel Oschin Comprehensive Cancer Institute, Cedars-Sinai Medical Center, Los Angeles, 90048 CA USA; 2 Department of Statistics, The Ohio State University, Columbus, 43210 OH USA

**Keywords:** Metagenomics data, Networks analysis, Modules

## Abstract

**Background:**

The human microbiome plays a critical role in human health. Massive amounts of metagenomic data have been generated with advances in next-generation sequencing technologies that characterize microbial communities via direct isolation and sequencing. How to extract, analyze, and transform these vast amounts of data into useful knowledge is a great challenge to bioinformaticians. Microbial biodiversity research has focused primarily on taxa composition and abundance and less on the co-occurrences among different taxa. However, taxa co-occurrences and their relationships to environmental and clinical conditions are important because network structure may help to understand how microbial taxa function together.

**Results:**

We propose a systematic robust approach for bacteria network construction and structure detection using metagenomic count data. Pairwise similarity/distance measures between taxa are proposed by adapting distance measures for samples in ecology. We also extend the sparse inverse covariance approach to a sparse inverse of a similarity matrix from count data for network construction. Our approach is efficient for large metagenomic count data with thousands of bacterial taxa. We evaluate our method with real and simulated data. Our method identifies true and biologically significant network structures efficiently.

**Conclusions:**

Network analysis is crucial for detecting subnetwork structures with metagenomic count data. We developed a software tool in MATLAB for network construction and biologically significant module detection. Software MetaNet can be downloaded from http://biostatistics.csmc.edu/MetaNet/.

## Background

Our human body is a host to various of microbes. Over 90 % of the cells in human body are bacterial or other non-human cells. These microbes have great influence on human physiology and nutrition, and are crucial for our health [1]. Metagenomics, which is the study of genetic material recovered directly from uncultured microorganisms, has accelerated the analysis of functional biodiversity relevant to its ecology. The objectives of human microbiome research are to explore the host-microbiota interactions, associate differences in microbial communities with differences in metabolic functions and diseases, and understand how microbiota changes may affect human health [1]. Massive amounts of metagenomic sequencing data have been generated with advances in next-generation sequencing (NGS) technologies. There are two NGS methods for metagenomics: whole metagenomic shotgun sequencing (WMGSS) and 16S rRNA gene sequencing. 16S rRNA sequencing is an amplicon sequencing method for identifying and comparing bacteria present within a given sample, while WMGSS comprehensively sample all genes in all organisms present in a given complex sample. The two techniques are quite different and intend for answer different biological questions. It has been shown that 16S rRNA sequencing contains hundreds of thousands of 16S RNAs fragments and is an efficient tool to infer bacterial communities, while WMGSS is mainly used for functional delineation and it is generally not deep enough to detect rare species in complex communities [2, 3]. In this paper, we infer network structures and taxa co-occurrence with 16S rRNA sequencing. By examining the relationship of genome structure and function across many different taxa with NGS data, the scope of microbiology and of microbial evolution studies has been greatly broadened, and the field of systems biology has emerged [4, 5].

There have been great strides in determining the taxonomical and functional contents of a sample in the last several years. Many software packages including MOTHUR [6], UniFrac [7], QIIME [8], and SILVAngs [9] have been designed primarily for the analysis of 16S rRNA sequencing data, while the other software packages including MEGAN [5, 10], Phymm [11], NBC [12] were developed mainly for shotgun metagenomic sequencing data. Those tools provide different approaches for the comparison of microbial communities with metagenomic sequence data. One output from some of the software is the abundance counts (sequence reads) for each taxa. These taxa abundance counts can be further analyzed to identify taxa and microbial communities that are associated with human diseases by comparing taxa counts from two or more groups with different disease status. Study of the link between characteristics of a microbiome and human disease is a active area of research. Current approaches such as MetaStats [13] and MetaDistance [14] mainly focus on variations in abundance across different clinical conditions, ignoring the interactions and structural variations among taxa. However, bacteria taxa do not act alone, rather they form part of large interacting (co-occurrence) networks and may function together. Variations in network structures and taxon interactions may be associated with disease status and clinical phenotypes [15–18]. Therefore network methods specifically designed for metagenomic count need to be developed.

Networks methods and graph theory have been widely applied to gene regulatory network construction with expression data [19–21]. Network analysis has also proved powerful for studying the characteristics of metabolic networks and their impact on various functional and evolutionary properties [22–24]. RNA-Seq is a NGS approach to transcriptome profiling. It provides a far more precise measurement of levels of transcripts and their isoforms than other methods [25]. Local Poisson graphical (log-linear) model and Bayesian generalized graphical model for network construction have been developed with RNA-seq data recently [26, 27]. However, the log-linear model is not valid when there are zero counts or measures in the data, which is common in metagenomics. Also, the Bayesian Poisson graphical model is slow when the network size is large. It usually takes hours to construct a network with hundreds of nodes. Those parametric methods can not be applied to metagenomic count data without modification. Moreover, even though there are a few methods available for network construction with microbiome data [28–32], most methods for network analysis are based on pairwise correlations (or distance) and ignore high-order correlations. However, high-order (partial) correlation has the advantage over pairwise correlation, because it measures the conditional dependency between two taxa given the effect of other taxa being removed or fixed, and reflects direct correlation between taxa and excludes the between-taxon dependency due to other taxa. In addition, variance heterogeneity and non-normality of metagenomic count data make standard correlations invalid (e.g. Pearson correlation). One way to deal with the problem is to use proportion and log-ratio transformations [33]. However the log-ratio is not defined when there are zeros in the data and approximation methods have to be used.

In this paper, we propose a nonparametric approach for co-occurrence network construction and subnetwork structure detection. We propose similarity (or distance) measures between taxa derived by adapting distance measures between samples with abundance counts defined in ecology [34]. We also expand the sparse inverse covariance method to sparse inverse of general similarity matrices for high-order correlation. The performance of our methods are evaluated through simulation and publicly available metagenomic data sets. The proposed methods are efficient for detecting true network structures. Even though the co-occurrence network is just a description analysis from temporal snapshots, it may be informative regarding how microbial taxa function together.

## Methods

Given samples with or without associated phenotypes, our goal is to study the connectivity and subnetwork structures of bacteria taxa with human microbiome. The final output from 16S rRNA sequencing of the host’s microflora is an integral, non-negative number of sequencing reads for each taxon. Such reads are the metagenomic counts represented as

$$ X =\left[ \begin{array}{llll} x_{11} & x_{12} & \ldots & x_{1m} \\ x_{21} & x_{22} & \ldots & x_{2m} \\ \vdots & \vdots & \ddots & \vdots \\ x_{n1} & x_{n2} & \ldots & x_{nm} \end{array} \right]  $$

where X is the count matrix with n samples and m taxa, and *x*_*ij*_ denotes the total number of reads of taxon *j* in sample *i*. In case there have been known disease status or clinical conditions (**y**) available, we will also discuss methods for detecting structural variations across clinical conditions. Constructing a human metagenomic network requires several sequential steps: (i) estimating pairwise similarity (or distance) measures between different taxa, (ii) adjacency matrix construction, (iii) network structure (module) detection and differentiated networks. We will discuss each of these steps.

### Pairwise similarity measures

Correlation coefficients are one type of similarity measures that describe the magnitude and direction of association between two variables. Because metagenomic count data typically have variances that are a function of the mean and are not normally distributed, the usual pairwise correlation (e.g. Pearson correlation coefficient)is not appropriate for network analysis. We use two distribution-free nonparametric correlations for count data. Given two n-dimensional vectors **x** and **y** and their corresponding ranks **R**_*x*_ and **R**_*y*_, we have

- Spearman rank-order correlation:
  $$R (\mathbf{x}, \mathbf{y}) = 1 - \frac{6\sum_{i=1}^{n} (\mathbf{R}_{xi} -\mathbf{R}_{yi})^{2}}{n(n^{2}-1)} $$
  We will take the average of the scores when multiple elements have the tied ranks.
- Kendall’s *τ* rank correlation:
  $$\tau(\mathbf{x}, \mathbf{y})= \frac{\sum_{i< j} \text{sgn}(x_{i} < x_{j}) \text{sgn}(y_{i} < y_{j})}{\sqrt{(T_{0} -T_{1})(T_{0} -T_{2})}}, $$
  where *T*_0_=*n*(*n*−1)/2, $T_{1} = \sum _{k} t_{k} (t_{k} -1)/2$, and $T_{2} = \sum _{l} u_{l} (u_{l}-1)/2$. The *t*_*k*_ is the number of tied **x** values in the *k*th group of the tied **x** values, *u*_*l*_ is the number of tied **y** values in the *l*th group of tied **y** values, and sign (*z*) is defined as:
  $$\text{sgn}(z) = \left\{ \begin{array}{rl} 1 & \text{if} \,x_{i} < x_{j}, \\ 0 & \text{if} \,x_{i} = x_{j}, \\ -1 & \text{if} \,x_{i} > x_{j}. \end{array} \right. $$

Our similarity matrix *S* can be defined with either $S = \left [\sin \left (\frac {\pi }{2}R\left (\mathbf {x}_{i}, \mathbf {x}_{j}\right)\right)\right ]_{m\times m}$ or $S = \left [\sin (\frac {\pi }{2}\tau (\mathbf {x}_{i}, \mathbf {x}_{j}))\right ]_{m\times m}$ [35]. Those distribution-free correlations only utilize rank information, and are more robust than the parametric approach. Even though they are slight less efficiency than Pearson correlation under normal distribution, both Spearman and Kendall correlation coefficients provide a good compromise between robustness and efficiency [36].

Distance measures are commonly used for quantifying the dissimilarities between samples and visualizing the samples in 2D and 3D [37]. They have been modified to measure pairwise similarity between taxa and construct phylogenetic tree recently [38, 39]. Given two n-dimensional column vectors **x**_1_ and **x**_2_ of two taxa, distance measures between taxa can be defined as

- Hellinger distance:
  $$D(\mathbf{x}_{1}, \mathbf{x}_{2}) =\sqrt{\sum_{i=1}^{n}\left(\sqrt{\frac{x_{i1}}{\mathbf{x}_{+1}}} -\sqrt{\frac{x_{i2}}{\mathbf{x}_{+2}}}\right)^{2}}, $$
  where $\mathbf {x}_{+1} = \sum _{i=1}^{n} x_{i1}$, and $\mathbf {x}_{+2} = \sum _{i=1}^{n} x_{i2}$.
- The *χ*^2^ distance:
  $$D\left(\mathbf{x}_{1}, \mathbf{x}_{2}\right) = \sqrt{\sum_{i=1}^{n} \frac{\left(\mathbf{x}_{+1} + \mathbf{x}_{+2}\right)}{(x_{i1} + x_{i2})} \left(\sqrt{\frac{x_{i1}}{\mathbf{x}_{+1}}} - \sqrt{\frac{x_{i2}}{\mathbf{x}_{+2}}}\right)^{2}}. $$
- Bray-Curtis dissimilarity:
  $$D(\mathbf{x}_{1}, \mathbf{x}_{2}) = 1 - 2 \frac{\sum_{i=1}^{n}\min(x_{i1}, x_{i2}) }{\sum_{i=1}^{n}(x_{i1} + x_{i2})} = 1-2\sum_{i=1}^{n} \frac{\min(x_{i1}, x_{i2}) }{(\mathbf{x}_{+1} + \mathbf{x}_{+2})}. $$

These distances can be calculated with either raw or relative abundance reads. Even though there is no great difference, we suggest to use relative abundance for sequencing depth adjustment. The relative abundance matrix *P* is computed from the count matrix *X* with *P*= [ *p*_*ij*_]_*n*×*m*_, where $p_{\textit {ij}} = \frac {x_{\textit {ij}}}{\sum _{j=1}^{m} x_{\textit {ij}}}$. Based on the distance measures, we define a similarity measure with the popular Gaussion kernel as

$$S =\, [\!S_{ij}]_{m\times m}, \qquad \text{where} \qquad S_{ij} = S(\mathbf{x}_{i}, \mathbf{x}_{j}) = e^{-\frac{D^{2}(\mathbf{x}_{i}, \mathbf{x}_{j})}{{\sigma}}}, $$

where the free parameter *σ* can be estimated by resampling. We set *σ*=1 for all computations in this paper. This distance based similarity matrix *S* is a positive (semi)-definite kernel matrix well studied in machine learning and bioinformatics. The kernel function $e^{-\frac {D^{2}(\mathbf {x}_{i}, \mathbf {x}_{j})}{{\sigma }}}$ can be treated as an inner product in the high-dimensional feature space, so *S* can be regarded as the covariance matrix in the feature space.

### Similarity to adjacency matrix

To compensate for noise and measure error, we propose two efficient approaches to determine statistically significant nonzero similarities. Unlike most methods in the literature determining the network structure with an arbitrary threshold of pairwise correlation, we are more interested in studying high order correlations. i.e., how **x**_*i*_ and **x**_*j*_ associate with each other when information about other variables is taken into consideration. Sparse inverse covariance for graph construction was originally proposed for continuous data with the assumption that the observations are from a multivariate Gaussian distribution [40]. This approach can handle large network efficiently. We extend this method to study the sparse inverse of a general similarity matrix *S*^−1^, and evaluate its efficiency using simulation. Unlike *S*, a value zero in any cell of *S*^−1^ implies conditional independence among those variables. Mathematically $S_{\textit {ij}}^{-1} = 0 \Rightarrow P(\mathbf {x}_{i},\mathbf {x}_{j}|\mathbf {x}_{-i, -j}) = 0$, where **x**_−*i*,−*j*_ denotes all the variables other than **x**_*i*_ and **x**_*j*_. The likelihood estimate of *A*=*S*^−1^ is

$$\max_{A\succ 0} L = \log\det A - tr(SA), $$

where *tr*(*SA*) is the trace of *SA*. Assuming *S* is nonsingular, and taking the first order derivative, we have *A*^−1^=*S*. However, it is common that *n*<*m* in metagenomic data, so *S* can be singular. In such case, the following *l*_1_ penalized error function can be minimized to obtain maximal likelihood estimates:

$$ \min_{A\succ 0} E = -\log\det A + tr(SA) + {\lambda} ||A||_{1}, $$

where $||A||_{1} = \sum _{\textit {ij}} |a_{\textit {ij}}|$ is the elementwise *l*_1_ norm for matrix *A*. The sparse structure of A can be estimated directly. This approach follows the framework of block coordinate descent [40, 41]. Mathematically, we partition the matrices *S* and *A* into the following block form:

$$S =\left[ \begin{array}{ll} S_{11} & \mathbf{s}_{12} \\ \mathbf{s}_{12}^{T} & s_{22} \end{array}\right]; \qquad \qquad A =\left[ \begin{array}{ll} A_{11} & \mathbf{a}_{12} \\ \mathbf{a}_{12} & a_{22} \end{array}\right], $$

where $S_{11}, A_{11} \in \mathbb {R}^{(m-1)\times (m-1)}$, $\mathbf {s}_{12}, \mathbf {a}_{12} \in \mathbb {R}^{m-1}$, and $s_{22}. a_{22} \in \mathbb {R}$. Then we have

$$\begin{array}{@{}rcl@{}} \log\det A = \log\det \left[A_{11}\left(a_{22} - \mathbf{a}_{12}^{T}A_{11}^{-1}\mathbf{a}_{12}\right)\right] = \log\det A_{11}\, +\, \log \left(a_{22} - \mathbf{a}_{12}^{T}A_{11}^{-1}\mathbf{a}_{12}\right). \end{array} $$

So

$$\min_{A\succ 0} E = -\log\det A_{11} - \log (a_{22} - \mathbf{a}_{12}^{T}A_{11}^{-1}\mathbf{a}_{12}) + tr(SA) + {\lambda} ||A||_{1}. $$

Assuming *A*_11_ is fixed and taking the first order derivative for **a**_12_ and *a*_22_, we have the sub-differential of E with respect to **a**_12_:

$$\frac{\partial E}{\partial \mathbf{a}_{12}} = \frac{2}{a_{22} - \mathbf{a}_{12}^{T}A_{11}^{-1}\mathbf{a}_{12}}A_{11}^{-1}\mathbf{a}_{12} + 2\mathbf{s}_{12} + 2{\lambda}\text{sgn}(\mathbf{a}_{12}), $$

where $\text {sgn}(x) = \frac {\partial |x|}{\partial x}$ for $x\in \mathbb {R}$ is defined as sgn(*x*)={1 *x*>0,[−1,1] *x*=0,−1 *x*<0.Similarly, since *a*_22_>0, the partial derivative of *E* with respect to *a*_22_:

$$\frac{\partial E}{\partial a_{22}}= -\frac{1}{a_{22} - \mathbf{a}_{12}^{T} A_{11}^{-1}\mathbf{a}_{12}} + s_{22} + {\lambda}. $$

After finding the derivative, we initialize *A*^0^=(*S*+*λI*)^−1^, and then use the standard decent gradient algorithm to update each row/column repeatedly until the algorithm converges. After obtaining the sparse A and taking the absolute value *A*=|*A*|, we set the diagonal value of A to zero with *A*=*A*−diag(*A*) to get the final adjacency matrix A. The adjacency matrix *A* is a representation of a graph, where the value of *a*_*ij*_ represents the connectivity between taxa *i* and *j*.

## *λ* Determination

The regularization parameter *λ* controls the number of nonzero estimated links between nodes and the sparsity of the network. The larger the *λ*, the sparser the network. A common approach for determining *λ* is stability selection [42, 43]. This approach seeks the *λ* leading to the most stable sets of edges. Given data *X*, stability selection first draws *p* sub-samples *X*_1_, *X*_2_,…,*X*_*p*_ of size q (1<*q*<*n*), where $q =\frac {2}{3}n$ in this paper, and then estimates one separate network *A*^*i*^(*λ*) for each sub-sample *X*_*i*_ and a fixed regularization parameter *λ*. Stability selection then defines the average fraction of disagreements over all edges of the sub-sampled graphs as

$$ D({\lambda}) = \frac{\sum_{j<k}\bar{a}_{jk}({\lambda})(1-\bar{a}_{jk}({\lambda}))}{{p\choose2}}, \;\; \textrm{where }\;\; \bar{a}_{jk} = \frac{1}{p}\sum_{i=1}^{p} a_{jk}^{i}({\lambda}). $$

The optimal $\hat {{\lambda }}$ is then chosen as:

$$\hat{{\lambda}} = \min\left\{{\lambda}: \max_{0 < t < {\lambda}}D(t) \le \alpha\right\}, \;\;\; \text{where}\;\;\; \alpha = 0.05. $$

Final network is constructed using whole data *X* and $\hat {{\lambda }}$.

### Network structure detection and differentiated networks

The problem of subnetwork structure detection requires the partition of a network into communities (subnetworks/modules) of densely connected nodes, while nodes belonging to different communities are sparsely (weakly) connected. Bacterial subnetwork structure detection is very important because we want to know which taxa coexist and function together. One simple approach to accomplish this is modularity function maximization [44, 45]. The modularity function also measures the quality of a partition and can be used to compare the performance of different partition methods. Given a weighted network with adjacency matrix *A*, to attribute each node to a module *c*_*i*_, a modularity function can be defined as follows:

$$ Q = \frac{1}{2w}\sum_{i,j}\left[a_{ij} -\frac{k_{i} k_{j}}{2w}\right ]\delta(c_{i}, c_{j}), $$

where *a*_*ij*_ is the weight of an edge between taxa (node) *i* and *j*, $k_{i} = \sum _{j} a_{\textit {ij}}$ is the sum of the weights of edges attached to taxon i, and $w = \frac {1}{2}\sum _{i,j} a_{\textit {ij}}$ is the total weight. In addition, *c*_*i*_ is the subnetwork (module) to which taxon i is assigned, and the *δ* function *δ*(*u*,*v*)=1 if *u*=*v* and 0 otherwise. Obviously, −1≤*Q*≤1 and the larger Q indicates better separation in subnetworks. The subnetwork partition algorithms are designed for maximizing Q. We adopt the two-step iterative local greedy approach [44] in this paper. Unlike K-means or hierarchical clustering, this two-step algorithm automatically determines the number of network modules (clusters) without predefining.

Given metagenomic data from different clinical conditions or different times, we construct a network with similarity *S*^*i*^ and adjacency matrix *A*^*i*^ for clinical condition or time i. We are interested in knowing wether the network structures of a subset of taxa have changed from one clinical condition (time) to another. We may define network statistics to measure the network structure changes either with the similarity matrix *S* or the adjacency matrix *A*. Given two networks *A*^1^ (*S*^1^) and *A*^2^ (*S*^2^), our first network statistic is defined by the following mean absolute distance (MAD):

$$\Delta(A) = \frac{1}{m(m-1)}\sum_{i<j<m}|a^{1}_{ij} - a^{2}_{ij}|, \; \text{or} \; \Delta(S) = \frac{1}{m(m-1)}\sum_{i<j<m}|s^{1}_{ij} - s^{2}_{ij}|, $$

where $a^{1}_{\textit {ij}}$ and $a_{\textit {ij}}^{2}$ are the interaction score between taxa *i* and *j* in network 1 and 2. The networks are considered to be significantly different if the value of *Δ*(*A*) or *Δ*(*S*) is large. Permutation tests can be used to estimate the *P*-value. Exact permutation test will be used when the sample size is small (<100), otherwise, the number of permutations used will be *L*=100, 000. We first permutate the original data *L* times and compute the *Δ*(*A*,*π*) or *Δ*(*S*,*π*) for each permutation *π*, the *P*-value corresponding to *Δ*(*A*) or *Δ*(*S*) can be computed as:

$$P(\Delta(A)) = \frac{1}{L} \sum_{\pi}I(\Delta(A,\pi)\ge \Delta(A)), \textrm{or }\, P(\Delta(S)) = \frac{1}{L} \sum_{\pi}I(\Delta(S,\pi)\ge \Delta(S)), $$

where *I*(*x*)=1 if *x* is true and 0 otherwise, and

$$\Delta(A, \pi) = \frac{1}{m(m-1)}\sum_{i<j< m}\left|a^{\pi, 1}_{ij} - a^{\pi, 2}_{ij}\right|, \text{or} \, \Delta(S, \pi) = \frac{1}{m(m-1)}\sum_{i<j< m}\left|s^{\pi, 1}_{ij} - s^{\pi, 2}_{ij}\right|. $$

### MetaNet package

MetaNet toolbox in MATLAB was implemented to construct sparse network from metagenomic count data. The toolbox was tested under MATLAB 2013a, but should also work on the later versions of MATLAB. Implemented functions in this toolbox include several similarity (distance) measures, simulated distributions and network models, sparse inverse covariance estimation, network structure detection and differential networks, and network visualization. The goal of this distribution is to provide an easy-to-use tool for network construction and analysis. Although the package is till under development, the users can construct, analyze, and visualize a network from their own data without much difficulty. MetaNet is provided as is without warranty of any kind. More information and the toolbox can be downloaded from http://biostatistics.csmc.edu/MetaNet/.

### Data sets

#### Simulated data

Simulated count data with different numbers of nodes and sample sizes are generated from a negative binomial (NB) distribution. More specifically, the data sets are generated from a NB distribution *X*_*ij*_∼*NB*(*λ*_*ij*_,*γ*) with mean *λ*_*ij*_ and dispersion parameter *γ*, and $\log ({\lambda }_{i})$ is from a multivariate distribution $\log ({\lambda }_{i}) \sim N(\mu, \Sigma)$ with mean *μ* and covariance matrix *Σ*. The graphical structures are constructed through *A*=*Σ*^−1^, where A is an adjacency matrix with additional diagonal elements $a_{\textit {ii}}=\sum _{j, i\neq j} a_{\textit {ij}}+1$, *i*=1,…,*n*. The adjacency matrices are generated using three different models include small world, scale free, and range dependent networks. The small world network we use only allows a node to connect with its neighborhood node, while the scale-free network has the number of nodes of degree 2 following a power law, and the range dependent network has an edge between nodes *i* and *j* with probability 0.9.0.3^|*j*−*i*|−1^. These three networks are known to mimic the behavior of real biological networks. All count data sets in this paper are generated by setting *μ*=3 and the overdispersion parameter *γ*=2.

#### Real metagenomic data from body habitats

The real data was collected from six body habitats including external auditory canal (EAC), gut, hair, nostril, oral cavity, and skin [46]. The objective of the original study was to estimate the microbial community composition and detect the differentiation in abundance among body habitats. A total of 815 samples were collected for 6 categories of habitat. Networks were constructed from gut, oral cavity (OC), and skin samples with the sample sizes of 45, 54, and 612 respectively. There were total 1713 taxa at the genus level.

## Results

### Results with simulation data

**Fig. 1:**
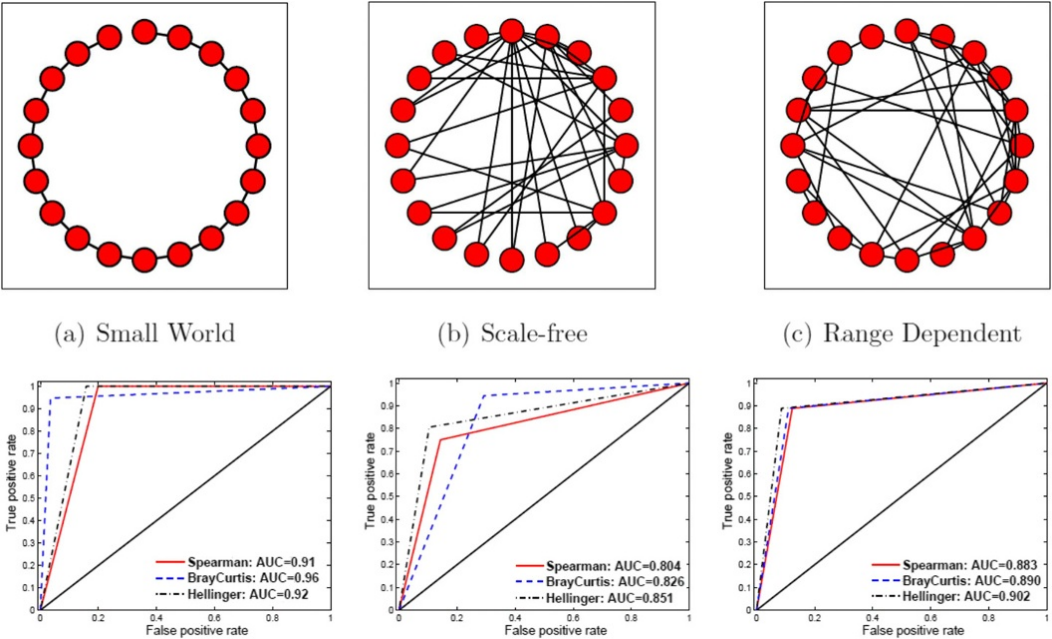
Simulated networks. Results from simulation data with three network structures: *Top* panel: three different network structures; *Bottom* panel: predicted AUCs for each simulated network

The proposed approaches were first evaluated using simulated count data. Given the number of nodes *n*=20 and sample sizes of *m*=500, we simulated the count data with known network structures. The predicted adjacency matrix was then estimated with the inverse of similarity matrix. The regularized parameter *λ* was chosen via stability selection. Computational results with different similarity measures and graphic models are given in Fig. 1.

The proposed similarity or dissimilarity measures including Spearman correlation coefficients, Hellinger, and Bray-Curtis performed well to detect the true structures as shown in Fig. 1. The area under ROC curves (AUC) was used to evaluate the performance of detecting proposed network structures, where the specificity for a network measures the proportion of no edges that are correctly detected, while the sensitivity for a network is the proportion of edges that are correctly identified. With the optimal *λ*^∗^=0.2,0.55, and 0.65 for Spearman, Hellinger, and Bray-Curtis, we have the predicted AUCs of 0.91, 0.96, and 0.92 respectively with the small world network. The Bay-Curtis distance performed the best, while the other two measures also performed well (≥0.91). Similarly, our proposed approach also performed reasonable well with both scale-free and range dependent networks. We achieved the best predicted AUC of 0.851 and 0.902 with Hellinger distance and *λ*^∗^=0.6 and 0.45 respectively for scale-free and range dependent networks. Overall Spearman has the lowest predicted AUCs for all models with the negative binomial simulated data as shown on the bottom of Fig. 1, but the differences among all measures are not very significant.

To further evaluate the performance of the method for large networks with small sample sizes and different similarity measures, Small world, scale free, and range dependent networks with 500 nodes and the sample size of 50,100, and 200 respectively are used for data simulation. The count data are generated from Poisson distribution with *μ*=3. We repeated the computational experiments 50 times, the average AUC and their standard deviations with different similarity measures are reported in Table 1.

**Table 1:** Predicted AUCs with different network structures and sample sizes for large networks with 500 nodes

*n*	Similarity	Small world	Scale-free	Range-dependent
50	Spearman	0.909 (±.015)	0.930 (±.035)	0.730 (±.021)
	Bray-Curtis	0.894 (±.014)	0.786 (±.017)	0.693 (±.016)
	Hellinger	0.936 (±.018)	0.844 (±.019)	0.709 (±.015)
100	Spearman	0.982 (±.009)	0.972 (±.020)	0.849 (±.014)
	Bray-Curtis	0.975 (±.011)	0.820 (±.016)	0.794 (±.019)
	Hellinger	0.986 (±.016)	0.883 (±.013)	0.812 (±.011)
200	Spearman	0.999 (±.012)	0.994 (±.015)	0.872 (±.015)
	Bray-Curtis	0.996 (±.009)	0.864 (±0.014)	0.828 (±.018)
	Hellinger	0.998 (±.006)	0.944 (±.012)	0.846 (±.015)

Table 1 indicates that the predicted AUCs increase and the performance gets better as the sample size increases. The proposed method performs reasonable well for different network structures. While Hellinger distance achieves the best result in small-world network, Spearman’s rank correlation has the best performance with scale-free and range dependent networks. Therefore, Spearman’s rank correlation is more robust with large networks generated from Poisson distribution, even though differences among different similarity measures are not always statistically significant.

### Results with real body habitats data

**Fig. 2:**
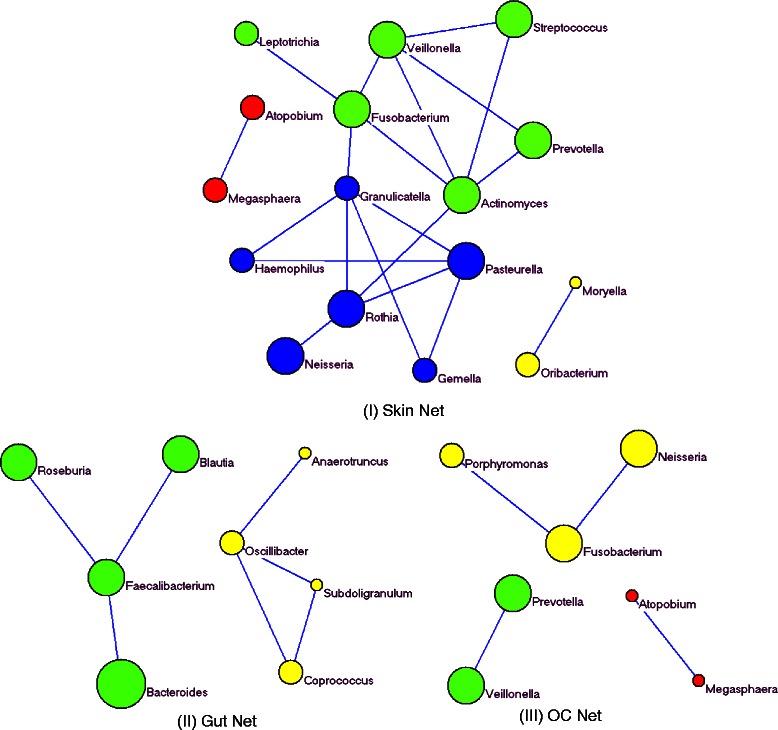
Differentiated networks for Skin (I), Gut (II), and OC (III)

There were 59 genera left for network construction after discarding genera with average abundance of less than two reads. We first calculated the similarity matrices from Spearman correlation coefficient, Hellinger, and Bray-Curtis distances respectively, and then determined the adjacency matrices with sparse inverse of the similarity matrices. The optimal *λ*s for Spearman, Hellinger, and Bray-Curtis were then determined with stability selection. To further reduce the false-positive rate, the final adjacency matrix *A* was determined by the common edges of three different adjacency matrices. For comparison purpose, we also constructed a common network with all the samples from different habitats. Exact permutation test was used to compare networks constructed from gut, oral cavity, and skin with the common network. The differentiated network structures for skin, gut, and oral cavity were detected with a permutation *P* value of 0.05. Subnetwork structures were then identified with modularity maximization. Bacteria networks unique for skin, gut, and oral cavity are shown in Fig. 2.

Different colors of the nodes indicate different network modules, and the node size represents relative abundance: The larger the nodes, the higher the relative abundance of a genera. The edges indicate the direct coexistence (co-occurrence) between two genera. The skin subnetwork on the top panel of Fig. 2 has 4 modules colored in green, blue, orange, and red with 6, 6, 2, and 2 genera respectively. Several genera on the network are known to cause skin infections. For instance, *Streptococcus* on the green module is a well-known bacteria directly related to several skin infections including Impetigo, Cellulitis, and Erysipelas. *Actinomcyes* genus causes a chronic (slowly progressive) infection named Actinomycosis, and Fusobacterium genus has been known to cause tropical phagedenic ulcer (http://dermnetnz.org/bacterial/). More interestingly, the direct association of *Actinomcyes* and *Fusobacterium* on the green module was verified by a recent study experimentally [47]. The mixture and co-infection of two bacteria genera cause mastoiditis. In addition, *Pasteurella* genus has also been shown to cause skin disease [48]. Other genera directly connected to *Pasteurella* including *Rothia, Granulicatella, Gemella*, and *Haemophilus* may function together biologically and clinically through ‘guilt by association’. Even though the co-occurrences among genera are only verified statistically, their biological and medical implications need to be further validated in a wet lab, our methods provide a guidance for investigators in their research.

The gut network constructed with 45 samples is shown in the bottom left panel (II) of Fig. 2. Two modules colored with green and yellow respectively are identified, each of them with 4 genera. All 4 high abundance genera and their interactions on the green module are associated with gut related diseases. *Faecalibacterium* and *Bacteroidetes* genera are both associated with type 2 diabetics and obeisity [49]. Another recently study also indicates that *Blautia* and *Faecalibacterium* vary together during antibiotic therapy [50], and it has been shown that both *Roseburia* and *Faecalibacterium* have lower abundance in patients with Crohn’s Disease compared with their healthy siblings [51]. All these studies support our results that *Faecalibacterium, Blautia, Roseburia*, and *Bacteroidetes* interact with each other. However, interactions among the 4 genera (*Subdoligranulum, Coprococcus, Anaerotruncus*, and *Oscillibacter*) with lower abundance in the yellow module have not been well studied, even though individual genus has been reported in recent literature.

Bacteria genera in oral cavity (OC) play a key role in mouth infections and periodontal diseases. Oral cavity network built with genera from 54 oral cavity samples is shown in the bottom right panel (III) of Fig. 2. Three modules colored with green, red, and yellow were identified with 2, 2, and 3 genera on each subnetwork. Interactions and co-occurrences are identified among both high abundance genera (*Fusobacterium, Veillonella*, Neisseria, Prevotella) and low abundance genera (Atopobium, and Megasphaera) in OC. Genera such as Fusobacterium, Neisseria, Porphyromonas, and *Prevotella* have been known to be significantly different in abundance with different clinical conditions and disease status [52, 53]. The co-occurrences between *Porphyromonas* and *Fusobacterium* on the yellow module has been verified through a mouse model experimentally [54]. However, co-occurrences among *Fusobacterium, Porphyromonas*, and *Neisseria* together have not been explored. One interesting finding is the common interactions between *Prevotella* and *Veillonella* and between *Megasphaera* and *Atopobium* in the oral cavity and skin, indicating that some interactions and co-occurrences may be shared at different body habitats. Therefore, co-occurrence network analysis with proposed approach is useful for determining novel biological interactions that may help to decipher the structure of complex microbial communities. It is also useful for systematically exploring co-existence patterns in big metegenomics data that standard tools may fail to detect.

## Conclusions

We have developed a systematic approach for constructing networks and detecting subnetwork structures with metagenomic count data. Our contributions are in two areas: (1) we adapt distance measures between samples from ecology to compute similarity between taxa, and (2) we extend sparse inverse covariance methods for Gaussian models to determine high-order interactions with general similarity matrices. Based on both simulated and real data, our method can identify true and biologically important interactions and associations with limited computational experiments. One advantage of our approach is that it detects the partial (high-order) correlations among the taxa. Unlike pairwise correlation, partial correlation measures the conditional dependency between two taxa given the effect of rest taxa being removed or fixed. Therefore, networks constructed from partial correlation usually have lower false positive connections than those from pairwise correlation. In addition, modularity function maximization for structure detection in MetaNet automatically determines the number of network clusters, while other popular approaches such as K-means and hierarchical clustering require the number of clusters or a cutoff point to be predefined. Even though MetaNet is slightly computational intensive when comparing to the popular pairwise approach, it only takes minutes to construct a network with thousands of nodes. While our method has been developed for 16S rRNA sequencing data from human body, it can be applied to 16S sequencing data from other organisms. It may also be used to analyze whole metagenomic shotgun sequencing or RNA-seq, as long as the sequences are properly aligned. Note that our method constructs networks solely based on their statistical similarity or dissimilarity among taxa, the biological significance of the results has to be further validated in a web lab. Future works wledge and pathway information into our network constructions.

## Appendix: network statistics

Given the adjacency matrix *A* and the their subnetworks, different statistics has been defined to describe the network and taxa. The most important network statistics are [55]:

- Degree: number of links connected to a taxon (node) i, $k_{i} = \sum _{j} a_{\textit {ij}}$.
- Number of triangles around a taxon i: $T_{i} = \frac {1}{2} \sum _{j,k}a_{\textit {ij}}a_{\textit {ik}}a_{\textit {jk}}$.
- Clustering coefficient: Measuring the segregation of a network, $C = \frac {1}{m}\sum _{i}C_{i} = \frac {1}{m}\sum _{i}\frac {2T_{i}}{K_{i}(K_{i}-1)}.$
- Participation coefficient of taxon i, $Y_{i} = 1 - \sum _{m\in M}\left (\frac {K_{i}(m)}{K_{i}}\right)^{2}$, where *k*_*i*_(*m*) is the within-module degree of taxon i in module *m*.

We will apply network statistics to rank the bacteria taxa. The larger the network statistics, the stronger connectivity the taxa, and the more important the taxa statistically. The biological importance of those taxa with larger network statistics can be further validated in the lab.
